# Glasgow Royal Infirmary

**Published:** 1829-01-01

**Authors:** 


### GLASGOW ROYAL INFIRMARY.

### I. FRACTURES.

We resume the Review of Dr. Anderson's Report in the Glasgow Medical Journal. In the course of the quarter, five patients were admitted with fracture of the neck of the femur, four of whom had the symptoms usually denoting it to occur within the capsular ligament. Two of the latter were put upon the double-inclined plane, one with Dessault's splint, and the other with Hagedorn's apparatus; but in none could extension be kept up so long as to obtain the re-union of the bone. In the case, in which the fracture was external to the capsular ligament, Dessault's splint was used, and very good union was obtained; though the patient was upwards of 70 years of age. The following observations are indicative of much good sense, and form a sufficient answer to those who assail Sir Astley Cooper so longly and loudly on the subject of "non-union."

"Without entering on the controversy regarding the re-union of fractures within the capsular ligament, I may state, that I have dissected several cases of this kind, at different periods after the injury, in which I found no deposition of callus, but rather an absorption of bone; whilst in other cases, exterior to the joint, I have uniformly found a free deposit of new bone. The extreme difficulty of maintaining the necessary extension, and preventing the motions of the pelvis for so great a length of time, is acknowledged by the advocates of re-union, even under the most favourable circumstances of private practice. But the feeble constitutions of the patients, and the debilitating effects of protracted confinement in a public hospital, tend greatly to increase this difficulty. I am convinced, that if a given number of persons, above 60 years of age, without any fractures, but with constitutions such as poor persons at this age generally possess, were laid in bed in a crowded hospital, and had their limbs, and almost their whole bodies immoveably fixed in one position for the number of months required by the advocates of successful re-union, a great majority of them would either sink under the treatment, or require the machinery to be removed, from local excoriations, or sloughs, or from constitutional disturbance. By placing the limb, however, on a slightly double-inclined plane, firmly supported by pillows, and attending to the health for a few weeks, we shall generally enable our patients to walk out of the hospital with the assistance of a crutch, and occasionally without even the need of a stick."

Fractures, of the femur we suppose, are generally treated in the Glasgow Infirmary with the long splint, and extended position, a plan, which, we think, will be commonly found to answer the best. We shall give the particulars of one or two interesting cases.

CASE 1.—*Fracture of the right femur, apparently combined with an ancient fracture of the cervix.—Death.* Adam Aitchison, ætatis 74, was admitted 29th March, eight days after having fractured the right thigh obliquely in its middle third, by falling out of bed, to which he had been, for two years, confined by paralysis. On bringing the fractured ends into apposition by extension, a shortening remained to the extent of two inches and a half, accompanied with the symptoms that indicate dislocation on the dorsum ilii. Besides these, unusual thickening was felt in the neighbourhood of the trochanter; and on inquiry, it was found that, two years before, he had fallen on the right trochanter, since which time the shortening of the limb had obtained. From the history and symptoms, it was thought to be old fracture of the neck of the thigh-bone, with consequent shortening and deformity.

The limb was put up in straight splints, but sloughing taking place on the nates, it was necessary to lay him on his side. Though the back got well, the old man gradually sank, and died in little more than a month after his admission.

Dissection disclosed a very curious and interesting process. There was ununited fracture of the cervix femoris in the capsular ligament, the surfaces smooth, and the neck of the bone, almost completely absorbed. Nature, however, had endeavoured to compensate for this, by propping up the joint with a quantity of osseous matter between the trochanters, and extending a process of solid bone from them, to the junction of the rami of the ischium and pubes, on which its end rested. In this situation, a new joint had been formed, which admitted of limited motion. The recent fracture was ununited, but surrounded with callous formation.

In a fracture of the olecranon, put up in the usual way, bony union was obtained in little more than four weeks. This was the second case of the kind which occurred to Dr. Anderson in the course of the present year; "but, in both, the subsequent motion was for some time so painful and imperfect, that I consider the ligamentous union to be preferable." Fracture of the neck of the humerus is treated at the Royal Infirmary, by one long splint on the outside of the arm, a small pad of tow in the axilla, and firmly bandaging the limb to the side of the chest. Long bags filled with *bran*, are preferred to every sort of padding, as a defence against the splints, an invention which, according to Dr. A., has incalculably improved the treatment of fractures. Tow-padding, however, is stitched up by a nurse or attendant in a minute, whilst *bran* we should think would require a good deal of care and trouble to prevent its escaping from the case. This objection is suggested with all due humility, as Dr. A. has tried the plan, and we have not.

### II. HERNIA.

In a case of inguinal hernia, occurring in a child of nine months old, an approach to syncope was induced by eight leeches to the tumour, and the warm bath; after which the reduction was effected by the taxis. The tumour was large and tense, and strangulation had been present three hours. Dr. Anderson believes that, though seldom of use in the herniæ of adults, leeches are much to be depended on in cases like this. They should be freely employed, attention being paid to the subsequent bleeding. The time for the employment of leeches, in the adult, is *after* the operation, and applied to the abdomen. We are sure, that if bleeding and local depletion were more generally put in practice, they would serve to arrest, or moderate, at any rate, the not unfrequently fatal inflammation of the peritoneum.

### III. DISPLACEMENT OF THE VERTEBRÆ.

In a case of spinal injury, where the 10th, 11th, and 12th dorsal vertebræ projected backwards, extension was made from the shoulders and feet, and the luxated vertebræ pressed strongly forward with the knee. No sudden reduction took place, but on examination it was found, that the prominence had nearly disappeared. Sensation and heat, which were previously feeble, almost immediately returned in the extremities, though paralysis of the limb, the bladder, and the rectum, remained. The sequel of the case is unknown, the patient being removed from the hospital by his friends.

"I believe, that this man would have made a good recovery. The return of sensation and heat, even without any motion, seems generally to remove the risk of death from sloughing of the nates, &c.; and the reduction of the displaced vertebræ, in this case, was the undoubted cause of the immediate amendment that took place in this respect."

We remember seeing one case, that of a boy, where extension was had recourse to. He remained in the hospital for many months, and was removed, at the end of that time, certainly improved in his condition. In the following instance, the restoration of muscular motion was effected by proper and persevering treatment.

*Case* 2. A collier, ætatis 38, was felled to the ground, by the roof of a coal pit falling on his back; and, three days after the accident, was received in the Royal Infirmary. The power of motion in the lower extremities was lost, but heat and sensation remained; the belly was swollen; the bladder and rectum paralysed; the urine, when drawn off, bloody, and containing much puriform matter. On examining the back, the two last dorsal vertebræ were found to be protruded, and the integuments swollen above them; there was pain, but no crepitus on pressure. Before his admission, he had been leeched and bled, and, after it, the treatment consisted in purging with colocynth and croton pills, blistering, cupping, and moxas to the part, the sores from the latter, being finally changed to pea-issues. An increase of power was generally obvious after each application of the moxa, whilst he, also, received benefit from dry frictions, passive motion of the limbs, and the internal use of the cuprum ammoniatum. At the end of two months, the urine had become natural, he could command the sphincter ani, and, though still complaining of weakness in the loins and dysuria, could walk through the ward with but little assistance. Sometime after his dismissal, he shewed himself almost entirely recovered. Another case of a similar description, caused by slipping the foot with a heavy weight upon the head, was treated in the same way with the same good success.

### IV. Ulcers.

Most of the ulcers on admission were more or less irritable, inflamed, or even sloughy. The treatment, in the first instance, was rest, emollient poultices and fomentations, purging, leeching, &c. till a clean and granulating surface presented itself. Then the milder ointments, adhesive straps, nitrate of silver in substance or solution, the weak nitric acid or chloride of lime lotion, were employed. When the surface of the ulcer is healthy, but indolent, and the granulations pale, we have found "Bates's camphorated wash" an excellent application. When the granulations are small and covered with a glairy, dark secretion, the red precipitate, dusted on lint, and then its coarser parts blown off, is of use in obtaining a healthier surface. For sores inclined to slough, the compound tincture of benzoin is an excellent application. Lint dipped in water cold or tepid, as best agrees with the feelings of the patient, is frequently an useful, always a cleanly kind of dressing. The lint should be folded once or twice, and laid upon the sore, whilst oiled silk is put over it to prevent evaporation.

### V. Hospital Gangrene.

Three cases of this formidable malady occurred in one ward in succession, all of them a consequence of recent wounds. The ward was, at the time, unavoidably crowded, although there were no extra beds at the time.

"The first instance was that of a healthy young woman from the country, who had been bitten in the leg by a dog. The teeth penetrated the fascia, and gave rise to acute inflammation beneath it, for which it was found necessary to make incisions with the scalpel. The inflammation was relieved, and the incisions were healing, but gradually became inflamed, and covered with yellow sloughs, by the separation of which, the disease extended to the sound parts, so as, at last, to form a large sore.

"The second was also a young and previously healthy woman, who was admitted with acute cellular inflammation of the leg and foot, for which a free incision was made with the scalpel. The usual relief followed, and the incision had nearly closed, when, without obvious cause, and with no apparent constitutional affection, it gradually became inflamed and sloughy. The edges were everted, and the whole sore filled with a dirty yellow fungus, which appeared as if undergoing the process of being squeezed out of it. One slough of this kind succeeded another, and after each separation, the fascia and muscles were exposed, and the circle of the sore extended.

"The third case was a middle-aged woman, of rather intemperate habits, who occupied the next bed to the former. This had likewise been a case of cellular inflammation of the leg, from cold caught in her employment as a washer-woman. The disease was speedily checked by an incision, which was healing for a week after, at which time the edges became inflamed and everted, and the whole surface cast off repeated and deep sloughs, exactly like the former.

"The treatment was various, consisting chiefly of leeching, opiate fomentations and poultices, carrot poultices, cinchona powder soaked with ol. terebinthinæ, solution of sulphate of quina, and several other lotions. None of these had more than a temporary effect. A solution of muriate of antimony, and a solution of arsenic, were the only applications of real service. Both acted in the same way, viz. by detaching an eschar, and leaving a healthy surface beneath. The butter of antimony acted the more speedily, but both were equally effectual. I have no doubt that the actual cautery would have served still better; but these cases all healed without it."

### VI. URINARY ABSCESS.

The modern practice of making free incisions in cases of urinary abscess, has incalculably lessened the dangers of this affection, serious at the best. In a case at the Royal Infirmary, where a gangrenous spot had invaded the bottom of the scrotum, the following treatment was successfully employed. A gum catheter was passed, though not without difficulty, into the bladder, and a pint and a half of high-coloured urine drawn off, when the instrument was allowed to remain in the bladder. The abscess was then freely laid open by an incision, extending from the slough in the scrotum to the verge of the anus, evacuating a large collection of very fetid matter, whilst smaller incisions were likewise made in different directions. Extensive sloughs were thrown off, of course, and an abscess, situated deep towards the sacrum, afterwards laid open. Healthy granulations soon made their appearance; in the fifth week, a small fistula only remained, which was touched with the actual cautery and speedily healed up.

We now arrive at the conclusion of the "Report," previous to which, a word or two remains on the subject of operations. He seems to have been an exceedingly sensible man, who pulled off his hat to the statue of the Olympian Jove, remarking that he knew not how soon his divinity might again get a footing in the world. How many an author has rung the knell of the flap operation, and yet its exhumation, or rather resurrection, would seem to be at present far from problematical. Dr. Anderson observes that "all the greater amputations were performed by the double flap, with Lisfranc's knife, and I coincide with my colleagues in *preferring this operation to the circular incision*, except, perhaps, in the leg. For some time I was apprehensive that muscular fibres laid over the sharp end of a divided bone would produce a conical stump from absorption, even after cicatrization had taken place; and I was led to this opinion from having seen a case of this kind, which had been amputated by the late Dr. Monteath. This, however, must have been accidental, for I never met with it again; on the contrary, I believe there were never better stumps formed than by the method now recommended."

Whether the circumstance was accidental or not in Dr. Monteath's amputation we cannot pretend to divine, but certainly the most conical stump which we ever observed, was after the flap operation on the thigh. The patient died, and presented, on death, depositions of matter in the cellular membrane, between the muscles of the limb, and some of those *traumatic* abscesses in the lungs. The objections to the operation are boldly, if not successfully, met by Dr. A.

"Dr. Ballingall's objection, that the muscles of the flap are sometimes longer than the integuments, is best obviated by a careful attention to the position of the limb at the time of operating, so as to prevent the division of the muscles whilst they are either relaxed or on the stretch. Even in the circular amputation of the thigh, the abduction of the limb, and the division of the triceps whilst in a state of extension, is the frequent cause of a sinus at the inner side of the stump, and of an uneven surface afterwards. The elongation of the large nerves and tendons before their division, in the flap operation, has sometimes made it necessary to remove parts of them with the scissors, before applying the dressings; but I believe this arises from bluntness of the knife, and the increased force which is thus required in carrying it outwards.

"The oblique division of the vessels, and the occasional difficulty in applying the ligatures from this cause, is less readily prevented, but does not form a serious objection where the parts are so much at command, as on the face of a flap. On the whole, the saving of pain to the patient, the greater facility of operating to the surgeon, and the expedition both in the operation and in the cure, induce me to think it probable that the circular incision will soon give place to the flap operation."

In this last prediction, we doubt but the author will prove a "false prophet," at least as regards the practice on this side the Tweed.

A *resurrection* man was received in the hospital with sphacelus of the foot, in consequence of exposure to cold in his *calling*. Amputation being necessary, was performed in what seems to have been a very simple and advantageous mode, viz. disarticulation of the metatarsal bone of the great toe from the cuneiforme magnum, and sawing in a line with this, the remainder of the metatarsal bones. A flap was brought from the sole of the foot, and a very neat stump thus formed. The patient, whose habit of body was bad, subsequently died with chronic bronchitis and emphysema of the lungs.

Before making our congé to Dr. Anderson, we beg to return him our best acknowledgements, as well for the pleasure as the profit we have reaped from perusing his report. Would to Heaven the example were more generally followed by our country brethren. Attached to public hospitals, possessed of much experience, and offered the means, in our medical journals, of recording that experience, with but trifling trouble, and no expense, why do they hang back, why bury in their graves, the little, or, perhaps, the important improvements suggested to their minds in the course of their career? The Emperor Tiberius is said to have wished that, when he died, both Heaven and Earth should expire with him! Our *Pythagorean* practitioners are Tiberius's in their way, immolating at their urns, not the material world indeed, but useful, and hard earned information. We trust that a better and a busier spirit is abroad.

### XLIX.

### ROYAL INFIRMARY, GLASGOW.

REPORT OF CASES TREATED IN THE SURGICAL WARDS, FROM 1st Nov. 1827, TO 1st MAY, 1828. By J. COUPER, M.D., Senior Surgeon.*

During the above-mentioned period, 199 patients were under treatment, ten of whom died—eight were dismissed with advice — seventeen relieved — one was turned over to the care of the physician —one was dismissed at his own desire—twenty-one still continued under treatment—and the rest were cured.

A table, displaying the nature of the cases, is appended, for which we refer to the original report.

1. ERYSIPELAS—EFFECT OF INCISIONS. —Erysipelas would appear the alpha and omega of surgical writings and surgical reports. It has ever been a subject of peculiar difficulty—it will ever be one of the keenest dispute, and no wonder, when we look to its varying forms—its dubious essence — and last, not least, epidemic character. A practitioner, from local circumstances, or other immaterial, perhaps unaccountable causes, sees much of a particular form of the disease. This form may be either the atonic or the tonic — the typhoid or inflammatory, in which case the surgeon very naturally becomes wedded to one mode of treatment, adapted, no doubt, to most of the cases he himself has seen, but totally misplaced in other situations, and at other times. The man who begins his professional career with the fixed determination to treat erysipelas by bark or by bleeding, will soon, if he carries his eyes in his head, discover what a narrow and fatal idea he has conceived. It may be that the nature of disease, like that of its subject—the human constitution, undergoes an inscrutable though still a decided alteration with the lapse of years; certain it is, that some modes of treatment once thought specific are at present pernicious in the gross. In the days of Dr. Fothergill, to take an illustration, bark was universally employed in erysipelas, apparently with better, infinitely better effects than would follow its general exhibition now. The taste of this day is rather for incisions, carried, at least in our humble opinion, to a very unnecessary if not an injurious extent. The disease is bad enough, but the remedy to bear is a thousand times worse. Incisions by the foot or by instalments are very pleasant subjects for a joke or a debate—the surgeon only makes them, but the patient has to feel them.

In making these remarks, we are far from underrating the value of the practice, we would merely protest against its indiscriminate employment. In far the greater number of cases it is totally uncalled for, and therefore, from the pain and suffering it produces, objectionable in the highest degree. In the acute and phlegmonous form of erysipelas none can be more favourable to incisions than ourselves, none more impressed with their absolute necessity. Another case exists, where early incisions are indispensably necessary for the safety of the limb or the life of the patient; where they act like a charm in arresting sloughing and procuring relief; and, in fact, where the favourable issue of the case hangs on their bold and judicious employment. A man receives an injury, we will say a compound fracture of the leg. In two or three days the limb, more especially in the neighbourhood of the wound, is observed to pit very slightly on pressure, and soon an emphysematous crackle is felt beneath the skin. The skin itself now assumes a dusky hue, which extends, and presents all the characters of the "brown erysipelas." If the surgeon is contented with bark and wine, the erysipelas, if such we may call it, extends, whilst the skin is extensively destroyed, and exposes beneath, vast putrid sloughs of bad and stinking cellular tissue. In this case, it is evident that the cellular membrane is affected first, the skin in only a secondary manner. If, then, incisions be freely made immediately the "emphysematous crackle" is felt, and before the integument is seriously affected, the latter will be sound, and the issue given to the putrid purulent matter and sloughs will prevent the extension of the disorganization of the cellular texture. To Mr. Brodie the profession are indebted for clearly pointing out the value of early and free incisions in cases of this description.

* Glasgow Medical Journal, No. IV.

To return from this digression, which we hope will be neither irrelevant nor useless, we learn from the report, that Dr. Couper's practice in "cutaneous erysipelas" consists in the free and repeated application of leeches, smart purges, antimonials, and evaporating lotions, with wine and tonics in subjects old or weak. In common "cutaneous erysipelas," we never saw much necessity for leeches, and therefore should seldom employ them. Moderate saline purgatives, evaporating lotions, and antimonials, are all that are in general required, at least in a city like London.

"Phlegmonous erysipelas, or diffuse inflammation of the cellular membrane, was uniformly treated by early and free incisions through the inflamed parts. Had former experience not convinced me of the propriety of this practice, the cases which occurred during the past winter, would alone have sufficed to demonstrate its advantages. I notice the following, because it affords an opportunity of comparing the effects of treatment with and without incisions."

*Case.* An officer of police, 43 years of age, was admitted on the 1st of December, 1827, with a sore ten inches in length and one in breadth, extending along the outside of the foot and around the outer ankle, its edges undermined, and its floor being a slough of yellow-coloured, dead cellular membrane. The whole of the integuments of the lower third of the leg were tense and greatly swollen, of a *dull red colour, and boggy to the feeling*, the back of the calf being covered with numerous vesicles. The strength was much reduced—the pulse 120 and small—the tongue brown—thirst great—occasional delirium. It appeared that, eleven days previous to admission, the patient had severely sprained his ankle; on the following day he had frequent rigors, and these were succeeded by inflammation of the integuments around the injured joint, and especially over the dorsum of the foot. Emollient and fermenting poultices were used, but the inflammation extended to the calf of the leg, vesications were formed, the skin became destroyed, and the patient was admitted in the state above-described.

The practice on admission consisted in an incision three or four inches long, completely through the inflamed integuments on the inside of the calf. The sore and wound were dressed with camphorated oil, and a bandage was lightly applied from the toes to the knee, that part of it covering the leg being constantly moistened with a lotion of spirits and lime water. The patient was instantly relieved, and no sloughing whatever took place on the leg, the inflammation of which had ceased at the end of a week. The slough on the dorsum of the foot came away, but the sore which it left was not healed till after a residence of nearly three months in the house.

Does Dr. Couper bring forward the above as an instance of the value of incisions in phlegmonous erysipelas? The passage we have quoted is all that immediately bears on the subject, and in it the language is somewhat ambiguous. It is evident that here was one of those cases of sloughing of the cellular membrane, attended with a dusky brown colour of the skin, rather than the florid and phlegmonous variety. It was not a severe example of the disease, but yet it proves distinctly the power of incisions. In the first instance none was attempted, and the cellular membrane sloughed to the extent of nearly a foot; in the second, an incision in the calf prevented the sloughing in that situation in toto. By the bye, we conceive that a poultice would have been a more proper application than a roller to promote the separation of the slough.

II. BURNS AND SCALDS.—In the excellent report from this institution, published in this Journal some time ago by Mr. Plymsoll, allusion was made to the good effects of dry cotton wool immediately applied to the injured parts. Dr. Couper, from experience, is inclined to think in a favourable manner of the practice.

"When the injury, however extensive, is of such severity as to produce only vesication, the cotton is by far the most advantageous application with which I am acquainted; and even in severer cases, when a portion of the integuments must slough, the patient may be saved from much of the debilitating discharge, which emollient and unctuous applications always produce, by the application of dry cotton, and the careful renewal of it, as soon as it becomes soiled with pus."

III. Hydrocele.—Since Sir James Earle introduced the practice of injecting for hydrocele, the modes which were formerly in vogue have been very nearly abandoned. Occasionally, though not very often, injection fails, and the surgeon is compelled to resort to other methods. In the case of an old man, in whom we saw the kali purum employed, a severe, and, indeed, if our memory serves us, a fatal attack of erysipelas succeeded. The practice is inferior to incision or excision of part of the tunica vaginalis. In a patient affected with hæmatocele, the tunica vaginalis was fully laid open by incision, and the cavity filled with lint. Abscesses formed in the scrotum, and smart constitutional disturbance ensued. In another case, that of a very large hydrocele, incision was performed, and the patient was many months in getting well. From these and other similar examples, our own impression is, that the above operations should never be performed in common hydrocele, unless the injection has totally failed, and that after several trials.

At the Glasgow Infirmary, four cases of this affection were treated by excision of a very small portion of the tunic, after the fluid had been let out by puncture. The operation was successful, but the inflammation which ensued was extremely severe. In one patient, indeed, fully one half of the scrotum sloughed, and many weeks elapsed before the testes were covered with granulations, and cicatrization of the wound had taken place. This mode of operating, therefore, says Dr. C. should only be had recourse to after injection has failed, a sentiment with which we cordially agree.

IV. Fatal Results of Extirpation of the Breast.—In one of the clinical articles in our last Number, we stated that we had seen a patient die of inflammation of the thorax, after amputation of the breast. The immediate result of the operation was an attack of erysipelas, and this was succeeded by the fatal thoracic affection. A patient of Dr. Couper's has lately died of pleuritis, supervening after the operation, and another from sloughing of the wound and exhaustion. We lately saw the life of a young woman, whose breast was removed for an encysted abscess, placed in the most imminent jeopardy by the supervention of erysipelas. These cases, and a number of others that might be mentioned, prove that the removal of the mamma is not so unaccompanied with danger as many would seem to imagine.

We are sorry our limits compel us to close our notice of Dr. Couper's report for the present. Much interesting matter remains behind, to which we shall return at another opportunity.

### LXVII.

### GLASGOW ROYAL INFIRMARY.*

#### 1. COMPOUND FRACTURES.

An instance of severe compound fracture of the superior maxillary, nasal, and malar bones, was treated by pressing them, as nearly as possible, into their natural situation, and dressing the wounds with adhesive plaster. By these means, with liberal bleeding, general and local, abstinence, and rest, suppuration was prevented, and the patient dismissed at the end of five or six weeks from the accident. The bones were consolidated, the wounds were healed, and scarcely any deformity remained. Dr. Couper observes, that fully alive as surgeons are to the bad effects of profuse discharge in compound fractures, they are little aware how often that profuseness depends on the frequent renewal of dressings. The following cases are adduced in illustration of the point.

*Case* 1. A lad of 16, had both bones of the right leg fractured obliquely, at the upper end of their lower third, by a kick from a horse. The wound of the integuments was small, and through it the fractured bones were felt—there had been some venous hæmorrhage—and the parts in the neighbourhood were swollen from ecchymosis.

"The limb was placed in the straight position, the wounds dressed with oiled lint, and splints and a bandage applied. During five weeks, these dressings were allowed to remain undisturbed, the patient suffering no pain in the limb, and not the slightest derangement of the general health; at the end of that time, on removal of the bandages, the bones were found completely united, the wound in the integuments still admitted the point of the little finger, but no bone could be felt either with the finger or a probe. The integuments above, and to outer side of wound, were undermined to the extent of about three inches, and, when pressed, discharged about two teaspoonfuls of healthy pus. About the same quantity of pus was found on the bandages. Immediately after this first dressing, pus was formed in such quantity, that the leg required to be dressed every day, or every second day, and many weeks elapsed before the undermined integuments became sound."

The second case is far more conclusive than the first. A boy was admitted on the 2d of April, with oblique fracture of the tibia, a little above its inferior third. The inner margin of the bone projected obliquely through a wound of the soft parts, and the integuments were detached for an inch around the wound. The limb was placed in the straight position, the wound dressed with dry lint and adhesive

* Glasgow Medical Journal, No. IV.

plasters, and a bandage and splints applied over all. The apparatus remained undisturbed till the 30th of April, when the bandage was removed, the integuments discovered to be sound, the bone united, the external wound the size of the base of a split pea. During the treatment no pain was experienced, and the health was undisturbed. A slight attack of dysentery detained the patient in the house till the end of the sixth week.

The above, though certainly instructive cases, are hardly, we think, fair illustrations of the absolute value of the practice pursued. Both were young patients, one, only eight years of age, and in these it is notorious that fractures are far less likely to produce those extensive suppurations and sloughings of the cellular membrane which constitute the harrassing and fatal sequelæ of compound dislocations and fractures in adults. The fact is, that the surgeon should be guided by the pain and the general disturbance produced. If these are inconsiderable, the less that the limb is meddled with the better; if great, the surgeon is a confident or careless man, who would suffer the dressings to remain undisturbed.

II. LARYNGITIS — BRONCHOTOMY PERFORMED WITH SUCCESS.—As Dr. Couper very justly observes, the propriety of opening the windpipe when an extraneous body is lodged therein is perfectly established. In dyspnœa, however, produced by other causes, the path is not so clear, nor the road so undisputed. The following cases bear upon the point.

*Case.* 1. A weaver, fifty years of age, was admitted on the 7th of November, 1827, with dyspnœa, and difficult deglutition. The voice was much impaired —the air during inspiration produced in its passage through the upper part of the larynx, a loud, moving noise, and, at times, a ringing sound—occasional paroxysms of violent cough, with copious but difficult expectoration of tough and yellowish sputum—parts in front, and at the side of the thyroid cartilage swollen and tender upon pressure, the swelling extending in a less degree towards the cricoid cartilage and os hyoides—no discoloration of the skin—nothing unusual in the fauces or the epiglottis—pulse 120, feeble, and thready — skin cold — aspect pale and haggard—strength much reduced. These were the symptoms, and it seemed that six weeks before, without obvious cause, the complaint began by swelling around the thyroid cartilage, followed by throbbing pain in the part. In the course of seven days the pain was relieved, but difficulty of breathing and swallowing commenced, and during the last eight days had been urgent.

Leeches, and after them a blister were immediately applied over the larynx, and a grain of calomel and the same amount of opium ordered to be taken every third hour. At nine, p. m. he was suddenly seized with dyspnœa so severe as to threaten immediate suffocation, and this was still so urgent when Dr. C. arrived, that he forthwith proceeded to open the windpipe. On account of the swelling above the larynx, the opening was made below the cricoid cartilage, which procured instantaneous relief for the dyspnœa. The wound was kept open by a bit of curved wire. No further difficulty of breathing took place, excepting that once a severe fit of coughing was caused by a little milk escaping through the wound. On another occasion, also, accidental derangement of the wire produced a slight paroxysm, but further than these, not the slightest inconvenience was felt after the operation. After a few weeks the wire was exchanged for a curved silver tube, about two and a half inches long, and one fourth of an inch in diameter, provided with two small rings, through which a piece of tape was passed, and tied round the neck, to retain the tube in situ.

"On the supposition that the contraction of the cavity of the larynx depended on thickening of its lining membrane, a mercurial course was prescribed, but apparently without benefit; for, although the patient continued to breathe easily, so long as the wound was kept open, yet all attempts to make him breathe through the mouth alone, proved ineffectual. On various occasions the wound was closed with adhesive plaster, to ascertain if any improvement had taken place, but it was invariably found necessary at the end of a few minutes to open the wound, and replace the tube, on account of increasing dyspnœa. At one time I entertained hopes of being able to dilate the contracted larynx, by bougies passed upwards through it from the wound; but the extreme irritability of the parts rendered this proposal impracticable. The introduction of even a probe through the wound into the larynx, was found to excite such a paroxysm of cough, that it was absolutely necessary to desist."

After remaining in the hospital above five months, the patient was dismissed, suffering no inconvenience except the necessity of breathing through the tube, a circumstance which habit had rendered very tolerable. By stopping up the tube with the point of his finger, he could speak in a hoarse, but audible tone. In the month of August last, he appeared at the Infirmary, and was perfectly free from all complaint.

*Case* 2. This was a tobacco-pipe maker, aged 28, who was seen by Dr. Couper, thirteen days after the commencement of the laryngeal inflammation. He had been bled pretty freely at different times, blistered, and treated with diaphoretic medicines, but still, though at times apparently relieved, the disease had proceeded on its march. When examined by Dr. C. he was just recovering from a fit of alarming orthopnœa, and presented these symptoms. Incapability of assuming the horizontal posture—inspiration laborious and wheezing—fauces red and swollen—epiglottis enlarged, tense, and shaped like a glans penis during erection—uneasiness decidedly referred to the larynx.

"Laryngotomy was immediately agreed upon. In making the incision through the teguments, a small artery was cut, and bled very freely. At the same instant the dyspnœa became greatly increased; the patient's face became livid, his limbs quivered, and his urine was ejected involuntarily. Without waiting to secure the artery, I immediately perforated the thyro-cricoid membrane, and the transition from the state now described to easy respiration was nearly instantaneous. The patient's body being inclined forward, no inconvenience was felt from the bleeding, which was speedily stopt by the pressure of the wire employed to dilate the aperture. From this time he continued to breathe easily, partly by the wound and partly by the mouth, and swallowed without difficulty."

Four days after the operation the wire was withdrawn, and on the 15th, the wound was so very nearly healed, that even during coughing no air escaped by it. Nine days after this, the patient had a rigor, followed by urgent orthopnœa, and a little pain and swelling of the right side of the larynx. After vainly employing a full dose of laudanum and antimonial wine, without relief, the larynx was opened a second time. In the course of ten days, the wire was changed for a silver tube which was kept in the wound for upwards of a month, and then withdrawn, shortly after which, the wound was healed. A few days after this, he was discharged, affected with only a glandular swelling on the left side of the neck, which soon disappeared on his leaving the hospital.

"Both of these cases appear important; the former as an example of contraction of the larynx produced by chronic inflammation, and the latter as an instance of the same effect arising from acute œdematous laryngitis. The important fact, that the dyspnœa, in cases of laryngeal disease, is liable to sudden and dangerous exacerbations, is well illustrated by both. Such paroxysms may cease, after the irritability of the parts is exhausted, but they will certainly recur again and again, until suffocation is produced, unless an artificial opening is made into the windpipe, to allow a free access of air to the lungs. When the necessity for it ceases, the aperture can be easily healed up; and even should the contraction of the larynx prove permanent, as in the case of Limpitlaw, it must be allowed, that the inconvenience arising from breathing through a tube inserted into the windpipe during the remainder of life, is small, when compared with loss of a limb, to which few refuse to submit as a mean of prolonging life."

---

